# Caveat emptor: for researchers, a rose will not smell sweet unless we know its composition

**DOI:** 10.1042/BSR20170078

**Published:** 2017-05-11

**Authors:** Josephine C. Adams

**Affiliations:** School of Biochemistry, University of Bristol, Bristol BS8 1TD, U.K.

**Keywords:** biglycan, decorin, neurite, SLRP

## Abstract

In a recent publication in Bioscience Reports “Contaminants in commercial preparations of ‘purified’ small leucine-rich proteoglycans may distort mechanistic studies”, Brown et al. identified by mass spectrometry and immunoblotting that certain commercial preparations of the small leucine-rich proteoglycans (SLRPs) decorin and biglycan, in fact, contained a mix of several proteoglycans that also included fibromodulin and aggrecan. The preparations were thus not suitable to study specific activities of decorin or biglycan. Decorin and biglycan are widely studied SLRPs that are considered to have highly multi-functional effects on cells. Decorin is of interest as a transforming growth factor-β antagonist and is also finding use in tissue engineering materials. This Commentary discusses Brown et al.’s findings and general issues raised for researchers who work with commercially sourced purified proteoglycans.

## The SLRPs

Brown et al. set out to study possible functional roles of the secreted, small leucine-rich proteoglycans (SLRPs) decorin and biglycan in supporting neurite outgrowth [1]. Proteoglycans are ubiquitous components of extracellular matrix (ECM) that are characterized by covalent substitution of one or more, typically *O*-linked glycosaminoglycan (GAG) chains on the core protein. Of the secreted proteoglycans of mammals, SLRPs form the largest gene family with the smallest core proteins (approximately 32–45 kDa) that are composed principally of a series of tandem leucine-rich repeats. Decorin and biglycan are closely-related SLRPs: their core proteins have 56% sequence identity in humans and are substituted with one or two chondroitin sulphate/dermatan sulphate-type GAG chains respectively (Table 1). Notwithstanding their small size, a wide array of functional properties have been attributed to decorin and biglycan, including roles in fibrotic diseases, infection and immunity, angiogenesis and tumour microenvironment. These properties relate to ECM-incorporated decorin and biglycan and also to activities of the proteins in soluble form. Correspondingly, an extensive set of interaction partners has been identified for each [2,3]. There is also interest in decorin as a tissue engineering material [4].

**Table 1:** Some characteristics of the major proteoglycans identified by Brown et al. [1] in commercial preparations of decorin or biglycan

Characteristic	PG			
	Biglycan	Decorin	Fibromodulin	Aggrecan
SLRP (Yes/No)	Yes	Yes	Yes	No
Mol. weight of core protein* (kDa)	41.7	38.8	43.2	250.4
Types and number of GAG chains	CS/DS (1 chain)	CS/DS (2 chains)	KS (4 chains)	CS/KS (multiple chains)

*Predicted molecular weights are based on the human proteins; CS, chondroitin sulphate; DS, dermatan sulphate; KS, keratan sulphate.

## The context

Both decorin and biglycan are expressed in intervertebral discs, which, in healthy individuals, are innervated only in the outer region. When discs degenerate, as occurs in ageing or after trauma, alterations in the disc ECM provide conditions that can allow for nerve ingrowth into central areas [5]. Although the most prominent phenotype of decorin-null mice is skin fragility [6] and, for biglycan-null mice, effects on growth of the long bones [7], both proteoglycans are known to affect motility and neurite outgrowth of neuronal cells and are up-regulated in the brain after injury [8,9]. Decorin promotes neurogenesis downstream of Wnt7a [10], and is of translational interest for reduction of scarring after spinal cord injury [11].

Given the presence of decorin and biglycan in intervertebral discs, the authors’ goal was to examine the activity of these SLRPs as substrata for neurite outgrowth by sensory neurons from dorsal root ganglia (DRG) explants. Their assay involved coating dishes with stripes of collagen I (an adhesive protein for neurons *in vitro*), interspersed with stripes of decorin or biglycan. The effects of decorin or biglycan, whether to support or repel neurites extending in from the collagen-coated strips, were then assessed microscopically. The DRG were from chick embryos and the proteoglycans were preparations from bovine articular cartilage, purchased from Sigma [1].

## The problem

As expected, the decorin and biglycan preparations inhibited neurite outgrowth, as measured by a concentration-dependent inhibition of entry of neurites on to the respective strips. However, warning bells were rung when treatment of the proteoglycans with chondroitinase AC, which cleaves chondroitin sulphate chains, failed to block inhibition. The authors entered into a series of control experiments. The identification of keratan sulphate in the preparations by immunoblotting with a highly specific antibody raised further concerns, because neither decorin nor biglycan is known to be substituted by keratan sulphate chains (Table 1). The authors, therefore, subjected the major protein bands detected after enzymatic removal of chondroitin sulphate, dermatan sulphate and keratan sulphate side chains to mass spectrometry analysis. Startlingly, biglycan was the most prominent protein in the decorin preparation: indeed the peptides from decorin did not meet the threshold for statistical significance. Consistent with the detection of keratan sulphate, fibromodulin and aggrecan were identified in both the biglycan and decorin preparations ( [1], Supplementary material) (Table 1). Finally, the authors immunoblotted the biglycan preparation for all the identified core proteins, in comparison with an independent preparation of aggrecan from post-mortem human intervertebral disc. Indeed, decorin, fibromodulin, aggrecan and high molecular weight keratan sulphate species were detected in the commercial biglycan preparation along with biglycan, whereas lumican (included in the tests as a protein not detected by mass spectrometry) was not detected. In the decorin preparation, biglycan and aggrecan were also detected by immunoblot. Thus, the preparations were found to be unsuitable for the study of specific properties of decorin or biglycan. The authors discuss their results with regard to lost time and resources and the implications for previous research publications from many laboratories in which these preparations have been used [1].

## Any roses anywhere?

Researchers who work with these preparations will benefit from the full list of protein components identified by mass spectrometry. Could the co-isolated proteoglycans represent components of biologically relevant, multi-protein complexes from cartilage ECM? Although SLRPs are known as collagen-binding proteoglycans, and decorin is not registered as a binding partner of other SLRPs [3], native SLRPs from bovine nasal cartilage have been demonstrated to bind hyaluronate-Sepharose, suggesting a possible mechanism for indirect association with each other and aggrecan [12]. Furthermore, biochemical purification of proteoglycans from articular cartilage is typically a complex process, involving guanidinium hydrochloride extraction, followed by multiple fractionation steps including density-gradient centrifugation, then size-exclusion chromatography and/or ion-exchange chromatography. Even in such multi-step schemes, co-purification of SLRPs with each other or with aggrecan has been reported, for example, as in human articular cartilage [13].

## Further considerations

The present study gives warning to researchers using, or considering to use, commercial decorin and biglycan preparations from animal (usually bovine) tissues. Commercial sources for recombinantly expressed decorin and biglycan can provide alternatives. A recombinant preparation was used, for example, to study transforming growth factor-β antagonism by decorin in a spinal cord injury model [11]. Most suppliers express the proteins in tagged form for tag-affinity purification. The quality control for purity is usually SDS/PAGE and silver staining. Given that, in most cases, the SLRP is expressed in mammalian cells, it is not implausible that small amounts of co-purifying ECM proteoglycans, collagens or growth factors might be present. In addition, proteoglycans expressed in mammalian cells can show variability of GAG substitution, GAG length or sulphation, depending on the cell line and also within the same preparation [14,15]. Overall, mass spectrometry analysis remains an advisable control. Another solution could be to work with proteins expressed in insect cells because many proteoglycans of jawed vertebrates are not encoded in invertebrates [16]. Again, some commercial sources provide insect cell-expressed decorin. However, the addition of relevant GAG chains and/or GAG sulphation status could be a concern in these more evolutionarily remote systems. Chondroitin sulphate modifications are known to take place in insects ( [17] and references therein), but whether epimerization to dermatan sulphate is supported is less clear. Dermatan sulphate epimerase orthologues are predicted throughout deuterostomes, yet are not identifiable in insects or other arthropods (human NP_001074445 used as a BLASTP query). Sulphation of chondroitin sulphate chains upon SLRP expression in insect cells appears likely, as proteoglycan biosynthetic enzymes including sulphotransferases related to mammalian chondroitin 6-sulphotransferase or chondroitin 4-sulphotransferase are present in insects such as *Drosophila* [18]. Moving to a distinct expression system may also bring new confounding issues: High Five (BTI-TN-5B1-4) *Trichoplusia ni* insect cells, popular for baculovirus expression because of the high amounts of protein produced, secrete an endoglycosidase capable of removing chondroitin sulphate, at least from aggrecan [19].

The overall picture is that additional experimental modes, such as side-by-side testing of wild-type and decorin-null cells, gene editing, transcriptional silencing or viral expression of wild-type or mutants, must come to the fore to prove roles of decorin or biglycan in functional processes of interest. The reminder to ‘know your materials’ is timely in view of the current drive from researchers, institutions, funding bodies and publishers to enhance the transparency of research reporting.

## Abbreviations

DRG: dorsal root ganglia
ECM: extracellular matrix
GAG: glycosaminoglycan
SLRP: small leucine-rich proteoglycan
